# Liver Regeneration after Hepatectomy and Partial Liver Transplantation

**DOI:** 10.3390/ijms21218414

**Published:** 2020-11-09

**Authors:** Shintaro Yagi, Masaaki Hirata, Yosuke Miyachi, Shinji Uemoto

**Affiliations:** Department of Hepatobiliary, Pancreas and Transplant Surgery, Kyoto University, 54 Kawahara-cho, Shogoin, Sakyo-ku, Kyoto 606-8507, Japan; mhirata6341@kuhp.kyoto-u.ac.jp (M.H.); yo_miyachi@kuhp.kyoto-u.ac.jp (Y.M.); uemoto@kuhp.kyoto-u.ac.jp (S.U.)

**Keywords:** liver regeneration, hepatectomy, liver transplantation

## Abstract

The liver is a unique organ with an abundant regenerative capacity. Therefore, partial hepatectomy (PHx) or partial liver transplantation (PLTx) can be safely performed. Liver regeneration involves a complex network of numerous hepatotropic factors, cytokines, pathways, and transcriptional factors. Compared with liver regeneration after a viral- or drug-induced liver injury, that of post-PHx or -PLTx has several distinct features, such as hemodynamic changes in portal venous flow or pressure, tissue ischemia/hypoxia, and hemostasis/platelet activation. Although some of these changes also occur during liver regeneration after a viral- or drug-induced liver injury, they are more abrupt and drastic following PHx or PLTx, and can thus be the main trigger and driving force of liver regeneration. In this review, we first provide an overview of the molecular biology of liver regeneration post-PHx and -PLTx. Subsequently, we summarize some clinical conditions that negatively, or sometimes positively, interfere with liver regeneration after PHx or PLTx, such as marginal livers including aged or fatty liver and the influence of immunosuppression.

## 1. Introduction

As presented in the myth of Prometheus in ancient Greek mythology, the liver is an organ in the human body with a rapid and abundant regenerative capacity. Even when almost two-thirds of the liver is surgically removed, the remaining liver can rapidly restore its original size. In rodents, the remaining liver after 70% hepatectomy or 30% partial liver graft returns to a size of 100% in 7–10 days after the operation, with a peak in DNA synthesis in the first 3 days. In humans, restoration of the liver mass is complete around 3 months following right or left hepatectomy, with a peak in DNA synthesis at days 7–10 after the operation [1,2].

Liver regeneration is a highly orchestrated process influenced by various factors. In living donor liver transplantation, Haga et al. reported that the remaining left lobe of donors and left lobe graft of recipients regenerate more rapidly than the remaining right lobe of donors and right lobe graft of recipients [3]. Moreover, the regeneration rate is higher in the recipient’s left lobe graft than in the donor’s remaining left lobe [3]. They also showed that the recipient liver volume increased rapidly in the first 2 months, exceeding the standard liver volume, and then gradually decreased to 90% of the standard liver volume [3]. Using genetic fate mapping and a high-throughput imaging system of individual hepatocytes, Miyaoka et al. reported that the volume of hepatectomy affected the mode of liver regeneration; i.e., while the remnant liver following 30% partial hepatectomy (PHx) regenerated solely by hypertrophy without cell division, hypertrophy and subsequent proliferation almost equally contributed to regeneration after 70% PHx [4]. Moreover, although most hepatocytes entered the cell cycle after 70% PHx, only about half of the hepatocytes underwent cell division during liver regeneration, leading to an increase in hepatocyte ploidy [4]. These results suggest that liver regeneration is a flexible and variable process that depends on the condition of the remaining liver, graft, and metabolic environment of the host.

Liver regeneration is a very important topic not only for basic scientists but also for clinicians because PHx or partial liver transplantation (PLTx) is performed on the assumption that the liver would restore its original volume. In contrast, failure of liver regeneration leads to hepatic failure and death. Advances in molecular biology and vigorous research efforts have revealed various factors and signaling pathways associated with liver regeneration. Although this topic is very well treated [1,2,5,6,7,8,9,10,11], studies have focused mainly or only on animal models, and there are less clinical data in the area of human liver regeneration after a surgically induced damage (PHx or PLTx). We first provide an overview of the molecular biology of liver regeneration post-PHx or -PLTx. Subsequently, we summarize some clinical conditions that negatively, or sometimes positively, interfere with liver regeneration after PHx or PLTx, such as marginal livers including aged and fatty liver and immunosuppression.

## 2. Discussion and Literature Review

### 2.1. Liver Regeneration after PHx or PLTx

**Figure 1:**
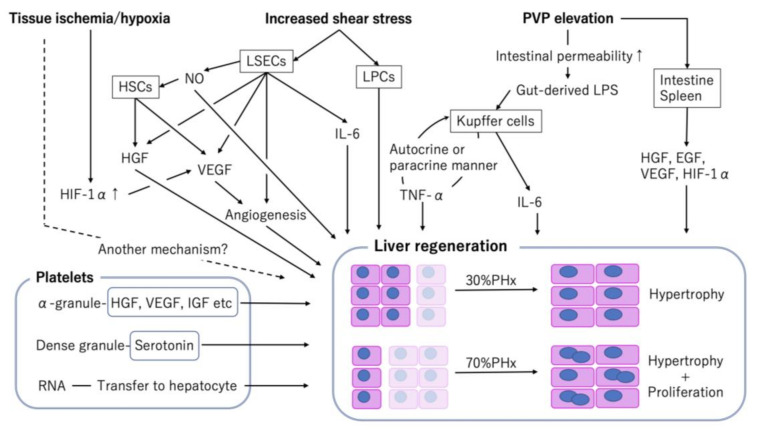
Factors associated with liver regeneration after partial hepatectomy (PHx) and partial liver transplantation (PLTx).Various factors are associated with liver regeneration after PHx and PLTx; moreover, the mode of regeneration is influenced by the amount of liver resection [4]. EGF, epidermal growth factor; HGF, hepatocyte growth factor; HIF-1α, hypoxia inducible factor 1-alpha; HSCs, hepatic stellate cells; IGF, insulin-like growth factor; IL, interleukin; LSECs, liver sinusoidal endothelial cells; LPCs, liver progenitor cells; LPS, lipopolysaccharide; NO, nitric oxide; PVP, portal vein pressure; TNF, tumor necrosis factor; VEGF, vascular endothelial growth factor.

Compared with liver regeneration after a viral- or drug-induced liver injury [12], that of post-PHx or -PLTx has several distinct features, such as hemodynamic changes in portal venous flow or pressure, tissue ischemia/hypoxia, and hemostasis/platelet activation (Figure 1). Although some of these changes also occur with liver regeneration after a viral- or drug-induced liver injury, they are more abrupt and drastic after PHx or PLTx. Hence, these can be the main triggers or driving forces of liver regeneration.

### 2.2. Hemodynamic Changes in Portal Venous Flow and Increase in Shear Stress

One of the most important triggers that initiate the liver regenerative process after PHx and PLTx is the frictional force applied by blood flow on the endothelial surface, the so-called “shear stress” [13]. After PHx or PLTx, the amount of portal venous blood flow per unit liver volume is inevitably increased since the portal venous flow consists of about 70% of inflow blood towards the liver [14]. This hemodynamic change affects the microstructure of the liver sinusoids. Within 6 hours following PHx, increased sinusoidal diameter, enlarged fenestration, disappearance of sieve-plate appearance, and widening of the inter-cellular spaces and space of Disse are observed, and it takes almost 10 days until the structure returns to its normal condition [15].

PHx drastically changes the blood flow to the liver, and this increases the shear stress on liver sinusoidal endothelial cells (LSECs). In response to increased shear stress, LSECs release nitric oxide (NO), which reinforces the hepatocytes’ sensitivity against hepatic growth factor (HGF) [16,17,18]. Inhibition of inducible or endothelial NO synthetase (iNOS and eNOS, respectively) severely suppresses liver regeneration after PHx in mice [19,20]. The iNOS and eNOS were thought to be associated with protection from cytokine-mediated hepatocyte injury and epidermal growth factor (EGF)-mediated hepatocyte proliferation, respectively [19,20]. Although some overlap may exist, other possible explanations about how NO affects liver regeneration include the following: (1) it has a balancing effect on cell apoptosis/proliferation; (2) it induces vascular endothelial growth factor (VEGF), which promotes angiogenesis, contributing to liver regeneration [21]; and (3) it stimulates hepatic stellate cells (HSCs) and promotes HGF and VEGF release [2].

Shear stress may also be associated with increased interleukin (IL)-6 release from LSECs. Kawai et al. showed that the increase in portal venous flow after portal venous embolization stretches the hepatic vasculature, possibly contributing to the increased levels of serum IL-6, a well-known hepatotropic factor, and regeneration of the non-embolized liver in humans [22]. Subsequently, they showed that mechanically stretched human LSECs released more IL-6 compared with non-stretched LSECs.

Liver progenitor cells (LPCs), which comprise less than 0.01% of parenchymal cells in adult livers, are known to have an abundant regenerative capacity during liver regeneration. Currently, Nishii et al. demonstrated that LPCs were also stimulated by increased shear stress and exhibited upregulation of several regeneration-related genes including *Wnt*, *VEGF*, and epithelial cell adhesion molecules [23].

### 2.3. Influence of Portal Venous Pressure (PVP) Elevation on Extrahepatic Organs

The contribution of increased gut-derived factors, such as lipopolysaccharide (LPS) on liver regeneration, has been well studied [10,24]. LPS, which is found in the outer membrane of Gram-negative bacteria and is a member of pathogen-associated molecular patterns (PAMPs), is recognized by pattern recognition receptors and elicits innate immunity [25]. PHx induces elevation of PVP and vascular shear stress, as well as intestinal permeability, which causes the increase of intestinal-derived PAMPs, including LPS. It has been observed that depletion of LPS negatively affects liver regeneration [26,27]. LPS is recognized by Toll-like receptors (TLRs) on Kupffer cells and triggers the release of IL-6 and tumor necrosis factor (TNF)-α [10], which are major hepatotropic factors promoting liver regeneration after PHx or PLTx.

The mechanism that triggers IL-6 or TNF-α release from Kupffer cells seems to be slightly more complex. Seki et al. and Campbell et al. found that mice lacking TLR2, TLR4, or TLR9, which are the major receptors recognizing LPS, had a normal IL-6 or TNF-α production after PHx [28,29]. Subsequently, Campbell et al. showed that knockout mice of myeloid differentiation factor 88, a common adaptor molecule required for signaling mediated by TLRs, showed severely decreased liver *Tnf* mRNA expression and circulating IL-6 levels after PHx compared with wild-type mice [29]. These results suggest that LPS and TLRs are not the only factors affecting Kupffer cells and that various factors and receptors may trigger the release of IL-6 or TNF-α from Kupffer cells.

Hepatotropic factors such as HGF, members of the EGF family, and VEGF are produced not only in the liver but also in extrahepatic organs surrounding the portal vein [30]. Using a rat PHx model, Yamamoto et al. demonstrated that the mRNA expression of several hepatotropic factors, including VEGF, HGF, HGF activator, and hypoxia-inducible factor-1α (HIF-1α), were also strongly upregulated in the intestine and spleen, in addition to being highly expressed in the liver [30]. Furthermore, the level of VEGF protein in the portal vein blood was significantly higher than that in the systemic circulation [30]. This means that extrahepatic organs may be important sources of hepatotropic factors and that the so-called “gut–liver axis” may play a crucial role in liver regeneration. This also suggests that some diseases or pathologies that disrupt the normal intestinal environment, particularly the gut microbiota, may negatively influence liver regeneration after PHx or PLTx [31].

Increase of shear stress or PVP is a “double-edged sword” in liver regeneration. In extended hepatectomy or liver transplantation using small-sized grafts, PVP or shear stress becomes too elevated, and this can cause hepatocyte injury and liver failure, which is called “post hepatectomy liver failure” [32] or “small-for-size syndrome” [33], manifested by large amounts of ascites, prolonged hyperbilirubinemia, and coagulopathy. Using a swine small-size graft liver transplantation model, we demonstrated that an early postoperative PVP elevation equal to or higher than 20 mmHg was associated with severe graft dysfunction and a poor outcome [34]. Considering that too little portal blood flow also causes graft dysfunction due to poor liver graft regeneration [35], there may be an optimal “range” of PVP or shear stress that initiates liver regeneration.

### 2.4. Liver Hypoxia

Liver hypoxia occurs as a result of either simple cessation of blood flow to the liver (Pringle’s maneuver or preservation time in liver transplantation) or decreased arterial flow due to portal hypertension of the remainder liver (arterial buffer response) after PHx [36,37,38]. Hypoxia is well known to be one of the strongest inducers of angiogenesis, and HIF-1α is known to play an essential role in hypoxic adaptation during mammalian development [39].

The expression of HIF-1α is suppressed under normoxic conditions. Once the tissue undergoes ischemia, HIF-1α is upregulated, forms a complex with HIF-1β, and binds the hypoxia response element, subsequently increasing the expression of genes, such as *VEGF*, erythropoietin, and glycolytic enzymes, allowing adaptation to hypoxic conditions [40,41]. Maeno et al. demonstrated that nuclear HIF-1α expression increased after PHx, followed by upregulation of VEGF and fms-like tyrosine kinase-1. Therefore, HIF-1α may be associated with sinusoidal endothelial reconstruction and promotes liver regeneration [42].

Recently, the influence of hypoxia on liver regeneration has gained strong attention in the context of associating liver partition and portal vein ligation for staged hepatectomy (ALPPS). Schadde et al. demonstrated that tissue hypoxia was higher in the portal vein ligation + hepatic transection (PVL+T) group than that of the PVL group [43]. Accordingly, the PVL+T group showed a higher HIF-1α-positive rate and enhanced liver regeneration than the PVL group [43]. Subsequently, they showed that aggravation of hypoxia in the PVL group promoted liver regeneration, whereas amelioration of hypoxia in the PVL+T group suppressed regeneration [43]. Using a rat model of ALPPS and portal vein ligation, Dirscherl et al. reported that hypoxia signaling by activated HSC via HIF-1α and subsequent angiogenesis play a crucial role in liver regeneration [38]. They proposed that HSCs act as hypoxia sensors in the liver and trigger angiogenesis during liver regeneration [38].

Although the promotion of liver regeneration by HIF-1α appears promising, it remains controversial. Ren et al. reported that hyperbaric oxygen preconditioning certainly increased the HIF-1α concentration in the rat liver; however, it did not strongly influence liver regeneration [44]. Furthermore, Kron et al. demonstrated that HIF-2α, and not HIF-1α, was associated with the upregulation of VEGF and proper induction of the angiogenic phase in the regenerating liver [45]. As the previously proposed effect of HIF-1α on liver regeneration is mediated by VEGF expression and angiogenesis, these results suggest that the influence of hypoxia on liver regeneration requires further elucidation and that there may be another mechanism that connects hypoxia and liver regeneration.

### 2.5. Factors Associated with Hemostasis

Hemostasis is an essential factor for a successful outcome after PHx or PLTx. However, growing evidence suggests that the factors associated with hemostasis are important not only for the cessation of bleeding but also for liver regeneration [46]. Among the various factors reported to be associated with liver regeneration [47,48,49], platelets undoubtedly play a pivotal role in the regenerative process. Several studies in both humans and rodents demonstrated that a low platelet count after PHx or PLTx was associated with poor liver regeneration and vice versa [50,51,52].

Lisman et al. summarized the potential mechanisms underlying platelet-mediated liver regeneration [53]. Following PHx, platelets rapidly migrate into the space of Disse and release their contents, stimulating hepatocyte or LSEC proliferation via HGF, VEGF, or serotonin [53]. Serotonin has a clear pro-regenerative effect on the liver [50], however, the exact and direct mechanism underlying this effect remained unclear for a long time. Recently, Fang et al. proposed that one way serotonin promotes liver regeneration may be via interacting with the Hippo signaling pathway (detailed later) [54]. Furthermore, some platelets are taken up by hepatocytes, which receive the functional RNAs of platelets, which enhance hepatocyte proliferation. It is known that platelets do not have a nucleus; however, they contain a wide array of RNAs, which can be translated into proteins. The in vitro study by Kirschbaum et al. demonstrated that platelet RNAs were transferred to hepatocytes, translated to functional proteins, and promoted liver regeneration [55]. Another possible mechanism is that platelets orchestrate an inflammatory response, i.e., by activating inflammatory cells, which then stimulate liver regeneration [53].

### 2.6. The Role of Bile Acid

The significance of the bile acid for the liver regeneration after PLTx and PHx is quite well studied at both the pre-clinical [56,57,58,59] and clinical levels [60]. The elevation of bile acid (salt) causes activation of the bile acid receptors on Kupffer cells and farnesoid X receptor (FXR) in the hepatocytes. FXR regulates cell cycle progression through Form1b, as well as through the FXR/fibroblast growth factor (FGF)19/FGF receptor (FGFR)4 signaling axis [59]. On the other hand, segmental cholangitis [61] and postoperative bile leakage [62] impairs liver regeneration after PHx. Moreover, choledochojejunostomy perturbs liver regeneration after PHx [58] and LTx [63] due to excessive inflammatory response in the liver and suppression of liver regeneration-associated factors in the clinical study.

### 2.7. Major Signaling Pathways Associated with Liver Regeneration after PHx and PLTx

**Figure 2:**
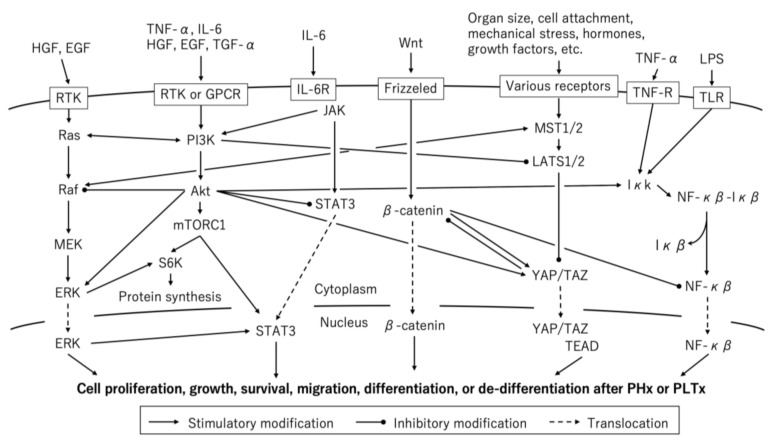
Molecular pathways associated with liver regeneration after partial hepatectomy (PHx) and partial liver transplantation (PLTx). EGF, epidermal growth factor; GPCR, G-protein-coupled receptor; HGF, hepatocyte growth factor; IFN, interferon; IL, interleukin; LPS, lipopolysaccharide; mTORC1, mammalian/mechanistic target of rapamycin complex 1; RTK, receptor tyrosine kinase; TGF, transforming growth factor; TLR Toll-like receptor; TNF, tumor necrosis factor.

The process of liver regeneration is usually divided into three phases: the priming/initiation phase, the progression/maintenance phase, and the termination phase. Various signaling pathways, receptors, and their ligands are involved in the complex process of liver regeneration. Furthermore, there is a complex crosstalk between these pathways, which sometimes shares a common route; therefore, it can be difficult to determine which pathway specifically regulates which phase of the cell cycle. However, this complexity means that there is no single nodal point in the process of liver regeneration that ultimately contributes to the host’s survival. In this section, in order to provide a concise overview of the regenerative process after PHx or PLTx, we summarized some of the major pathways involved (Figure 2).

#### 2.7.1. Ras/Raf/MEK/ERK Pathway

The Ras/Raf/MEK/ERK pathway is associated with cell cycle progression and transmits signals from growth factor receptors to downstream genes coding for several transcription factors, such as *c-myc, c-fos,* and *c-jun*. In liver regeneration, the pathway mainly regulates the initiation phase of the cell cycle. HGF is the main ligand of this pathway; in addition, EGF family members, fibroblast growth factor, or VEGF, which are ligands for receptor tyrosine kinases (RTK), also share the same signaling pathway [64]. Ras protein is a hub of complex cell proliferating pathways and interacts with phosphatidylinositol 3’-kinase (PI3K) [65]. Raf and ERK are also known to interact with other signaling pathways [65,66,67].

#### 2.7.2. Janus Kinase (JAK)/Signal Transducer and Activator of Transcription 3 (STAT3) Pathway

The JAK/STAT3 is a principal signaling pathway for liver regeneration. Activation of this pathway stimulates cell proliferation, differentiation, migration, growth, survival, and apoptosis. JAKs are activated through autophosphorylation, and further phosphorylate and activate STAT3, which translocates to the nucleus and acts as a basic transcription factor in liver regeneration. STAT3 is a very important transcription factor involved in the induction of gene expression via cytokines [68,69].

This pathway is primarily activated by IL-6 and its receptor, IL-6R. Recently, the importance of “IL-6 trans-signaling” on liver regeneration has gained considerable attention [70]. Usually, the IL-6/IL-6R complex binds to a second receptor protein, glycoprotein (Gp) 130, initiating the JAK/STAT3 pathway. Interestingly, cleavage of IL-6R by metalloprotease was found to form soluble IL-6R (sIL-6R), which could still bind to IL-6 and activate Gp130 [71]. Surprisingly, this phenomenon could also occur in cells without membrane-bound IL-6R. Fazel-Modares et al. showed that this “IL-6 trans signaling” is the main driver of liver regeneration following PHx and that classic signaling via membrane-bound IL-6R is insufficient to initiate the regenerative process [72].

#### 2.7.3. PI3K/Akt Pathway

PI3K is a ubiquitous protein kinase family involved in signal transduction through RTK or G-protein-coupled receptors [66]. Well-known PI3K receptor ligands in liver regeneration include TNF-α, IL-6, HGF, EGF, and transforming growth factor (TGF)-α [73]. When the receptor is activated, PI3K catalyzes the production of phosphatidylinositol-3,4,5-triphosphate, recruiting a subset of signal proteins including Akt. The phosphorylation of Akt ultimately leads to the activation of downstream molecules and expression of genes regulating cell growth, proliferation, or migration [73].

Genetic deletion of p85α, a subunit of PI3K, in mice leads to extensive hepatocyte necrosis, enlarged muscle fiber, brown fat necrosis, calcification of cardiac tissue, and ultimately perinatal death [74]. Inhibition of this pathway using wortmannin, a potent PI3K inhibitor, or small interfering RNA, markedly delayed liver regeneration and inhibited macrophage infiltration [73]. The compensatory effect of the PI3K/Akt pathway on the JAK/STAT3 pathway is confirmed. Using STAT3 knockout mice, Haga et al. demonstrated that PI3K/Akt-mediated hepatocyte hypertrophy compensated for liver regeneration [75].

Mammalian/mechanistic target of rapamycin (mTOR) is a serine/threonine kinase regulating various cellular functions, including cell proliferation and energy metabolism. mTOR is also an important downstream signal transducer of the PI3K/AKT pathway. Two distinct complexes of mTOR are found, namely, mTOR complexes 1 and 2 (mTORC1 and mTORC2), which differ in their composition, downstream targets, and sensitivity to rapamycin. Immunosuppressants, such as rapamycin (sirolimus) or everolimus, inhibit mTORC1 only. The two best known downstream effectors of mTORC1 are the eukaryotic translation initiation factor 4E-binding proteins and ribosomal protein S6 kinase; both enhance mRNA translation, thus promoting protein synthesis [76,77].

#### 2.7.4. Nuclear Factor-κB (NF-κB) Pathway

During the quiescent state, NF-κB is located in the cytoplasm and is inactivated by IκB. At the priming phase of liver regeneration, Kupffer cells are activated by LPS or TNF-α to further release TNF-α and IL-6. This process is regulated by the NF-κB signaling pathway. Once the receptors are activated, IκB is phosphorylated and removed by Iκk, allowing NF-κB to migrate to the nucleus, enhancing the expression of TNF, IL-1, IL-6, and VEGF [78,79]. The NF-κB pathway is also shown to have crosstalk with, for example, the JAK/STAT, PI3K/Akt/mTOR, and Wnt/β-catenin pathways [79].

#### 2.7.5. Wnt/β-Catenin Pathway

The Wnt/β-catenin pathway regulates cell proliferation, cell–cell adhesion, and tissue integrity, such as hepatic zonation during liver regeneration [80,81,82,83]. When the Wnt signaling is inactive, β-catenin is usually inactivated in the cytosol. Once Wnt binds to its cell surface receptor (Frizzled) and co-receptor (low-density lipoprotein–related protein 5 or 6), β-catenin escapes degradation and undergoes nuclear translocation, where it promotes the expression of target genes, such as *cyclin D1* or *c-myc* [84]. β-catenin was shown to interact with the NF-κB [85] and Hippo signaling pathways [8].

#### 2.7.6. Hippo Signaling Pathway

Recently, it was shown that the Hippo signaling pathway controls organ size via regulating cell proliferation, apoptosis, and stem cell self-renewal [86], and that it is the candidate pathway that manages controlled activation and cessation of hepatocyte proliferation during regeneration [87]. Indeed, the Hippo signaling pathway is rather a controller than a promoter of cell proliferation. For example, when cellular density is high, cell–cell contact produces a growth inhibitory signal via the Hippo signaling pathway. However, once the organ size decreases, this pathway promotes cells to exit the quiescent state and re-enter the cell cycle [88]. Yes-associated protein (YAP) is a main downstream effector of the Hippo signaling pathway and is usually inactivated by its upstream regulators mammalian Ste20-like kinases 1/2 (MST1/2) and large tumor suppressor 1/2 [88,89]. Once activated and translocated to the nucleus, YAP binds to the transcriptional enhanced associate domain and promotes the expression of its target genes [88]. YAP is a multifunctional protein also associated with regulation of the spindle assembly check point, cytoskeleton, or cell adhesion [90]. Various factors including organ size, cell attachment, mechanical stress, hormones, or growth factors can activate or inhibit this pathway [88,90,91,92]. Interestingly, vascular shear stress is also an activator of YAP and its paralog, transcriptional coactivator with PDZ-binding motif (TAZ) [93,94]. Oh et al. demonstrated that liver regeneration after PHx was reduced by half in mice with hepatocyte-specific deletion of YAP compared with that of the control group [95]. YAP or TAZ has crosstalk between the Wnt/β-catenin or PI3K/Akt pathway [96,97,98], although the detailed mechanisms underlying this have not yet been elucidated and the results are sometimes inconsistent.

### 2.8. Marginal Liver: Aged or Steatotic Liver

#### 2.8.1. Aged Liver

Aging is defined as the progressive loss of tissue and organ function [99]. Although the liver can regenerate in response to PHx in adulthood, its regenerative capacity gradually declines with aging [100]. Numerous factors have been proposed as the cause of impaired liver regeneration in aged animals [100]. In this section, we summarize the intracellular and extracellular elements of liver regeneration in aged animals.

Forkhead box M1B (FoxM1B) is a transcription factor expressed in embryonal cells that plays an important role in cell cycle progression [101,102]. Although its expression is usually limited to the period of embryonal development, FoxM1B is activated again during liver regeneration [101]. Wang et al. demonstrated that aged mice have decreased expression of FoxM1B after PHx and that this was associated with impaired liver regeneration [103]. Subsequently, they showed that old mice with transgenic overexpression of FoxM1B had augmented expression of cyclin D1 and cyclin B1 and restored regenerative capacity [103]. Likewise, CCAAT/enhancer-binding protein (C/EBP)α, a strong inhibitor of cyclin-dependent kinase, is also associated with age-related decline of liver regeneration. Iakova et al. demonstrated that aging elicits a change in the growth inhibitory activity of C/EBPα [96,97,98,99,100,101,102,103,104]. In young animal liver, C/EBPα is attached to cyclin-dependent kinase 2, maintaining the cells in a quiescent state [104]. Moreover, the age-related increase of Brm, a chromatin remodeling protein, enhances formation of the C/EBPα–Rb–E2F4 complex, which represses the *c-myc* promoter and leads to impaired liver regeneration [104]. Budding uninhibited by benzimidazole-related 1 (BubR1) is a multifunctional mitotic checkpoint protein that plays important roles in chromosome alignment on the mitotic spindle and in the spindle assembly checkpoint [105]. Ikawa-Yoshida et al. demonstrated that BubR1 expression was decreased in aged mice and that low-BubR1-expressing mutant (BubR1^L/L^) mice showed delay in liver regeneration compared to control mice [106]. Subsequently, they showed that BubR1 was associated with the expression of desmocollin-1, a type of cell adhesion molecule, and hypothesized that not only delayed hepatocyte proliferation but also detachment-induced cell death may be the cause of poor liver regeneration under low BubR1 expression [106]. Pibiri et al. demonstrated that while the hepatic expression of cyclin D1 was upregulated 24 h after PHx in young mice, it was profoundly downregulated in aged livers at the same time point [107].

Recently, YAP, which participates in the Hippo signaling pathway, has gained some attention as one of the important causes of age-related decline of liver regeneration [107]. Currently, Loforese et al. demonstrated that silencing of MST1/2, the kinase that inhibits activation of YAP, restored liver regeneration after PHx even in aged (12-month-old) mice [91]. Interestingly, they and Pibiri et al. found that YAP was paradoxically activated in aged mice even before PHx and remained high after PHx [91,107]. In contrast, activated YAP decreased significantly in aged mice with mitogen-induced enlarged liver before and after PHx. Therefore, Pibiri et al. proposed that YAP may be a sensor of organ size rather than a cell cycle regulator [107]. However, further studies are warranted to elucidate the involvement of the Hippo signaling pathway, including the function of YAP, in aged liver regeneration.

Several extra-cellular factors have also been shown to be associated with impaired regenerative capacity in aged liver, such as growth hormone, Src homology 2 domain-containing protein p66^Shc^, and interferon (IFN)-Υ [100]. Heterochronic parabiosis is a vascular anastomosis technique in which young and aged mice share the same blood circulation. Using this technique, Convoy et al. demonstrated that the expression of C/EBPα–Rb–E2F4 complex was reduced, and the regenerative capacity after PHx was restored in aged mouse liver, which was circulated with young mouse blood [108]. Liu et al. also showed that preoperative administration of young rat plasma restored the regenerative capacity of aged rats after PHx [109]. The results of these studies demonstrate that using young animal blood or plasma improved liver regeneration in old animals, however, liver restoration never reached the same level as that of younger animals. Therefore, their results suggest that a number of intrinsic pathways and extrinsic factors are involved in the altered mechanism of liver regeneration in aged animals.

#### 2.8.2. Steatotic Liver

Although it is expected that the steatotic liver will show impaired liver regeneration after PHx or PLTx, this is controversial. In clinical studies, for example, Ibrahim et al. showed that steatosis does not have a negative impact on liver regeneration; however, their studies were conducted in living donors with mild (less than 10%) steatosis [110]. On the other hand, Kele et al. demonstrated that patients with liver steatosis showed a trend toward worse liver regeneration after PHx [111]. However, as they also stated in the article, their study did not evaluate other factors that could affect liver regeneration, such as the preservation of the middle hepatic vein, the portal venous flow or spleen size [111]. Therefore, it remains unclear whether the impaired regeneration was simply caused by steatosis itself.

A similar controversy also exists in experimental studies. While some authors argued that steatosis caused impaired liver regeneration, others showed that the liver’s regenerative capacity was normal or even enhanced in liver steatosis after PHx (Table 1) [112,113,114,115,116,117,118,119,120,121,122,123,124]. This largely stems from the differences in the experimental protocol used, selection of liver steatosis model, the degree of fat accumulation, or evaluation method and the timing of liver regeneration. Among these, the most challenging is the choice of steatosis model. Interestingly, the results of studies conducted in early stage steatosis using a model of methionine- and choline-deficient diet (MCDD) exhibited almost the same degree of regeneration as that of normal liver [112,114,117]. In contrast, most of the genetically engineered liver steatosis models showed impaired liver regeneration after PHx [113,115,116,117,118,122]. Picard et al. found that among three different types of steatosis models, only fa/fa rats (genetically engineered leptin-deficient rats) showed impaired liver regeneration after PHx [117]. Several hypotheses have been proposed for this discrepancy. For example, it has been already known that a choline-deficient diet enhances the recruitment of LPCs in hepatectomized liver tissue [125]. Therefore, Diehl et al. proposed that this could be a factor promoting liver regeneration in this model [126]. However, their hypothesis was further questioned because now it is becoming clear that liver regeneration is largely achieved by hepatocyte proliferation, whereas the participation of LPCs is thought to be limited [127]. On the other hand, several authors proposed that defective leptin signaling rather than the presence of hepatic steatosis is the main cause of impaired liver regeneration, because leptin can enhance liver regeneration [117,126]. Furthermore, the same discrepancy can be found in other steatosis models. Tanoue et al. showed that while liver steatosis induced by a high fructose diet showed impaired regeneration, a high-fat diet-induced steatotic liver possessed a regenerative capacity that was comparable to that of normal liver [120]. They also suggested that altered gene expression profiles, in particular that of TGF-β1, may be attributed to differences in liver regeneration [120].

It should be noted that although the steatotic liver’s regenerative capacity is described as “not impaired”, it does not mean that it is the same as that of normal liver. For example, although the steatotic liver ultimately achieved the same degree of regeneration as that of normal liver, Rao et al. and Collin de l’Hortet et al. commonly found that the steatosis group showed a delay in the uptake of bromodeoxyuridine 5-bromo-2’-deoxyuridine (BrdU) at the early stage (<48 h after PHx) of liver regeneration [114,122]. Likewise, Zimmers et al. also reported a delay in liver regeneration manifested by liver mass and proliferating cell nuclear antigen (PCNA) expression [123]. Furthermore, Valdecantos et al. reported that despite impaired liver regeneration, BrdU uptake, and PCNA expression were paradoxically higher in the MCDD-induced steatotic liver 48 h after PHx compared to the levels in the control group [124]. Their results also suggested that BrdU uptake and PCNA expression might have already reached a peak and then decreased before 48 h after PHx in the control group. Therefore, the mechanism of steatotic liver regeneration is altered and impaired compared to that of normal liver.

Although the aforementioned controversies should be carefully considered, several molecular mechanisms affecting liver regeneration have been proposed. Selzner et al. demonstrated that obstacles during the cell cycle caused impaired liver regeneration in leptin-deficient ob/ob mice [113], whereas Collin de l’Hortet et al. showed that down regulation of the GH/EGFR pathway was associated with lower regenerative capacity in ob/ob mice [122]. Zimmers et al. also demonstrated that EGFR expression was downregulated in high-fat diet-induced liver steatosis and that the preoperative injection of an EGFR-expressing plasmid significantly improved survival after PHx [123]. Using a high-fat diet-induced liver steatosis model, DeAngelis et al. demonstrated that increased IκBα expression might be the cause of impaired regeneration after PHx [119]. As IκBα inhibits NF-κB activation, it could lead to decreased expression of its target genes, cyclin D1, and anti-apoptotic gene Bcl-xL [119].

### 2.9. Impact of Immunosuppressants

Liver-specific immunosuppression is required immediately after transplantation. Therefore, the impact of immunosuppressants on liver regeneration is a highly relevant topic, especially with regard to PLTx. Four kinds of immunosuppressants are commonly used in liver transplantation, namely, calcineurin inhibitor (CNI), mTOR inhibitor, mycophenolate mofetil (MMF), and steroids. However, the studies focusing on the influence of MMF or steroids on liver regeneration are relatively old and scarce compared with studies of the other immunosuppressants. In addition, the results of these studies are hampered by strong inconsistency [128,129,130,131,132,133,134], suggesting that MMF or steroids are likely to have very limited effects on liver regeneration. Hence, in this section, we focused on the influence of CNIs and mTOR inhibitors on liver regeneration.

#### 2.9.1. CNIs

CNIs, including cyclosporine A (CyA) and tacrolimus (also known as FK506), inhibit the function of calcineurin, which further suppresses the activation and nuclear translocation of a transcription factor called “nuclear factor of activated T cells”, which upregulates the expression of multiple cytokines and costimulatory molecules necessary for full activation of T cells [135].

Some reports demonstrated that CNIs augmented liver regeneration through both immunological and non-immunological mechanisms. Immunologically, the suppression of T cells and, in particular, of natural killer (NK) cells, by CNI is considered the reason behind enhanced liver regeneration [136,137] because NK cells secrete IFN-γ, which is the major aggravator of fulminant hepatitis [137]. Morii et al. demonstrated that TGF-β, a well-known inhibitor of liver regeneration that mainly acts during the terminal phase, was significantly decreased in CyA-treated rat liver [138]. Non-immunological effects of CNIs have also been suggested. Francavilla et al. demonstrated that tacrolimus enhanced liver regeneration in nude rats lacking T cells, showing that tacrolimus did not inhibit NK cell function in an in vitro study [139]. They hypothesized that FK-binding protein and cyclophilin, which are the target molecules of tacrolimus and CyA, have peptidyl-prolyl isomerase activity and enhance cellular growth [139]. On the other hand, several reports have demonstrated that CNIs have no or “at least” very limited tropic effects on liver regeneration [132,133,140,141]. Although the reason for these controversies remains unclear, it is similar to what has been discussed in the section on steatosis, that is, the different drugs used, administration period, evaluation method, and timing for liver regeneration.

#### 2.9.2. mTOR Inhibitor

Compared with CNIs, the clear advantage of mTOR inhibitors is the lack of nephrotoxicity and possible anti-cancer effect [142]. In contrast, the most worrisome disadvantage of mTOR inhibitors is the suppression of wound healing or liver regeneration. Rapamycin, which is the first mTOR inhibitor identified, has been reported to increase hepatocyte apoptosis and suppress liver regeneration during the first 5-7 days after PHx [131,143,144,145]. This is expected, because, as already described in the previous section, mTOR is a downstream effector of the PI3K/Akt pathway associated with cell growth, proliferation, and anti-apoptosis. The suppressive effect of mTOR inhibitors on liver regeneration is adamant. Fouraschen et al. reported that even treatment with IL-6 or HGF could not completely reverse the mTOR inhibitor-induced suppression of liver regeneration after PHx in mice [145].

Due to its suppressive effect on liver regeneration, it is not generally recommended that mTOR inhibitors be used during the early post-transplant period, especially in PLTx. However, as mTOR inhibitors lack nephrotoxicity and have anti-tumor effects, clinicians are encouraged to use them as early as possible after transplantation. Recently, several clinical studies revealed that mTOR inhibitors, including rapamycin and its precursor everolimus, can be started safely after 30 days following PLTx [146,147]. Importantly, mTOR inhibitors do not completely halt liver regeneration. Rupertus et al. reported that 12 days after PHx, the wet weight of the regenerated liver was comparable between the mTOR inhibitor-treated, CyA-treated, and control groups [148]. Therefore, in cases of non-small size graft liver transplantation, the time restriction of mTOR inhibitor use can be further eased in the future.

## 3. Conclusions

Liver regeneration is an essential factor for successful PHx or PLTx outcomes. In contrast, failed regeneration means postoperative liver failure, which is a great risk for the patient’s life. In this review, we focused on the intra- and extracellular molecular mechanisms underlying liver regeneration following PHx and PLTx. As described above, liver regeneration is a finely designed process involving a network of pathways and their interactions. Therefore, there is no single factor or pathway that can completely halt liver regeneration; if one pathway is blocked, another will compensate for it and achieve almost normal liver regeneration. Not even mTOR inhibitors, which are strong inhibitors of cell proliferation, can completely halt liver regeneration; they, instead, merely slow the process down [148].

Considering the complex and multi-layered network of pathways involved in liver regeneration, an old question emerges again—how does liver regeneration fail? The process of liver regeneration has been widely studied. However, liver regeneration failure studies are scarce. Nonetheless, we now have some indications. As described above, several factors that initiate liver regeneration after PHx or PLTx are, at the same time, inhibitors of liver regeneration. Too much portal vein flow or high pressure will cause regeneration failure [34]; similarly, long-lasting ischemia/hypoxia will lead to greater ischemia-reperfusion injury and subsequent liver failure [149]. Furthermore, metabolic demand from the host that is too high will also hamper liver regeneration [150]. Unlike portal vein flow or pressure, it is sometimes difficult to accurately quantify the metabolic demand of the host; however, bile acid is now considered a marker of decreased metabolic capacity [151]. However, bile acid, as well as portal vein flow or pressure, is a double-edged sword for liver regeneration. PHx drastically increases the bile acid/liver volume ratio, which, when it becomes too elevated, causes bile acid overloading in the remainder liver and impairs liver regeneration [8].

Failure of liver regeneration should be regarded as an all-out failure of the regenerating process or disruption of the pathway network system rather than a lack of a single hepatotropic factor or blockade of a single molecular pathway. This suggests that it may be difficult to rescue a failing liver by upregulating a single hepatotropic factor, molecular pathway, or transcriptional factor. Advances in non-surgical treatments urge surgeons to attempt more difficult surgical procedures, such as extended hepatectomy or liver transplantation using small-sized, aged, or steatotic liver grafts. Aging and liver steatosis, both common problems in contemporary society, have a negative impact on liver regeneration [99,152]. Therefore, elucidating the process of liver regeneration and readdressing the old question “how does the liver regeneration fail and how can we rescue it?” are now more important than ever.

## Figures and Tables

**Table 1:** Summary of experimental studies on liver regeneration after partial hepatectomy (PHx) of steatotic liver (separate file).

	Author	Published Year	Animal	Induction of Steatosis	Degree of Steatosis *	PHx	Evaluation of Liver Regeneration	Timing of Evaluation	Liver Regeneration
1	Zhang et al. [104]	1999	rats	MCDD for 4 weeks	Not mentioned	68%	Liver weight, [3H]-thymidine incorporation	24 h	Not impaired
2	Selzner et al. [105]	2000	rats	fa/fa rats, 20 weeks old	Severe	70%	Liver weight, mitotic index, BrdU, PCNA	24, 48, 96 h	Impaired
3	Rao et al. [106]	2001	rats	Choline-deficient diet for 4 weeks	Severe	70%	Mitotic index, BrdU	24, 36, 48, 72, 96 h	Not impaired
4	Yang et al. [107]	2001	mice	ob/ob mice, 8-10 weeks old	Severe	70%	BrdU, PCNA	24, 36 h	Impaired
5	Torbenson et al. [108]	2002	mice	ob/ob mice, 10 weeks old	Moderate	70%	BrdU	24, 36 h	Impaired
6	Picard et al. [109]	2002	rats	fa/fa rats, 13 weeks old	Moderate	70%	Liver weight, BrdU, PCNA(WB)	24 h	Impaired
				Orotic acid for 4 weeks	Severe				Not impaired
				MCDD	Severe				Not impaired
7	Yamauchi et al. [110]	2003	mice	db/db mice, 10 weeks old	Severe	70%	Liver weight, mitotic index, BrdU, PCNA	24, 48, 72, 120, 168, 240 h	Impaired
8	DeAngelis et al. [111]	2005	mice	High-fat diet, for 8-12 weeks	Severe	70%	Liver weight, BrdU	12, 24, 36, 48, 60, 72, 96, 120 h	Impaired
9	Tanoue et al. [112]	2011	rats	High-fructose diet for 4 weeks	Mild	70%	Liver weight, PCNA	24, 72, 168 h	Impaired
				High-fat diet for 4 weeks	Severe				Not impaired
10	Sydor et al. [113]	2013	mice	Western diet for 6 weeks	Mild	70%	Ki67, PHH3	24, 48, 168 h	Enhanced
11	Collin de l’Hortet et al. [114]	2014	mice	ob/ob mice, 13-14 weeks old	Severe	55%	Liver weight, mitotic index, BrdU, PHH3	32, 44, 56, 68 h	Impaired
				MCDD for 5 weeks	Severe				Delayed
12	Zimmers et al. [115]	2017	mice	High-fat diet for 11-13 weeks	Not mentioned	70% and 80%	Liver weight, BrdU, PCNA(WB)	6, 24, 48, 72, 96, 120 h	Impaired
13	Valdecantos et al. [116]	2017	mice	MCDD for 3 weeks ^§^	Moderate	70%	Liver weight, BrdU, PCNA(WB)	48, 336 h	Impaired ^‡^
				High fat diet for 13 weeks ^§^	Mild				Impaired

*: The degree of steatosis was subjectively assessed as mild: <30%, moderate: 30–60%, severe: >60%. ^§^: Special diet was continued after hepatectomy. ^‡^: BrdU uptake and PCNA expression were higher at 48 h after hepatectomy. BrdU, bromodeoxyuridine 5-bromo-2’-deoxyuridine; MCDD, methionine- and choline-deficient diet; PCNA, proliferating cell nuclear antigen; PHH3, phospho histone H3 staining.

## Abbreviations

ALPPS	associating liver partition and portal vein ligation for staged hepatectomy
BrdU	bromodeoxyuridine 5-bromo-2’-deoxyuridine
BubR1	budding uninhibited by benzimidazole-related 1
C/EBP	CCAAT/enhancer-binding protein
CNI	calcineurin inhibitor
CyA	cyclosporine A
EGF	epidermal growth factor
EFGR	epidermal growth factor receptor
eNOS	endothelial nitric oxide synthetase
eIF4E	eukaryotic translation initiation factor 4E
FGF	fibroblast growth factor
FXR	farnesoid X receptor
GH	growth hormone
Gp	glycoprotein
HGF	hepatic growth factor
HIF-1α	hypoxia-inducible factor-1alpha
HSCs	hepatic stellate cells
IFN	interferon
IL	interleukin
iNOS	inducible nitric oxide synthetase
JAK	Janus kinase
LATS1/2	large tumor suppressor 1/2
LPCs	liver progenitor cells
LPS	lipopolysaccharide
LSECs	liver sinusoidal endothelial cells
MCDD	methionine- and choline-deficient diet
MMF	mycophenolate mofetil
MST1/2	mammalian Ste20-like kinases 1/2
mTOR	mammalian/mechanistic target of rapamycin
NF-κB	nuclear factor-κB
NK	natural killer cells
NO	nitric oxide
PAMPs	pathogen-associated molecular patterns
PCNA	proliferating cell nuclear antigen
PHx	partial hepatectomy
PI3K	phosphatidylinositol 3’-kinase
PLTx	partial liver transplantation
PRRs	pattern recognition receptors
PVL+T	portal vein ligation + hepatic transection
PVP	portal venous pressure
RTK	receptor tyrosine kinase
sIL-6R	soluble IL-6 receptor
S6Ks	S6 kinase
STAT	signal transducer and activator of transcription
TAZ	transcriptional coactivator with PDZ-binding motif
TEAD	transcriptional enhanced associate domain
TGF	transforming growth factor
TLRs	Toll-like receptors
TNF	tumor necrosis factor
VEGF	vascular endothelial growth factor
YAP	Yes-associated protein
4E-BPs	eIF4E-binding proteins
